# Supplementary material for: MicroRNAs as Predictors of Future Uterine Malignancy in Endometrial Hyperplasia without Atypia
Source: J Pers Med. 2022 Feb 18;12(2):311. doi: 10.3390/jpm12020311 (PMC8879120; doi:10.3390/jpm12020311)
Supplement: Supplementary file 1 [file jpm-12-00311-s001.zip › jpm-1544059-supplementary.pdf]

Table S1. Demographics and clinical features of patients whose SH /CH-nonA and subsequently progressing to EC.

Study Code	SH Date	SH/CH Dx	Em. ca. Date	Histological Dx	Stage	Grade	MiRNA array/ QPCR/
SH-01P	1989/4/13	Focal polypoid hyperplasia	2004/5/11	endometrioid	IIIC2	3	MiRNA array/ QPCR
SH-02P	1998/4/27	SH without atypia	2004/2/27	endometrioid	IA	2	QPCR
SH-03P	2000/10/9	SH without atypia	2003/8/23	endometrioid	IA	1	MiRNA array/ QPCR
SH-06P	2008/9/26	SH without atypia	2009/4/14	endometrioid	IA	1	QPCR
SH-07P	1992/11/20	CH without atypia	2005/8/23	endometrioid	IA	2	MiRNA array/ QPCR
SH-08P	2008/8/26	SH without atypia	2012/4/24	endometrioid	IA	2	MiRNA array/ QPCR
SH-012P	2001/10/29	SH/CH without atypia	2009/6/15	endometrioid	IA	1	QPCR
SH-013P	2005/7/6	SH without atypia	2015/9/8	endometrioid	IA	1	MiRNA array/ QPCR
SH-014P	2011/4/1	SH/CH without atypia	2015/5/26	endometrioid	IA	1	MiRNA array
SH-15P	1996/5/14	SH without atypia	2005/7/27	endometrioid	IA	2	QPCR
SH-16P	2002/6/22	SH without atypia	2014/10/29	endometrioid	IB	1	QPCR
SH-17P	1996/4/17	SH without atypia	2004/12/31	endometrioid	IA	1	QPCR
SH-18P	2011/3/24	SH/CH without atypia	2017/4/25	endometrioid	IIIA	1	QPCR
SH-19P	2000/10/11	SH without atypia	2015/4/2	endometrioid	IA	1	QPCR
SH-20P	2003/10/23	SH without atypia	2012/11/15	endometrioid	IA	1	QPCR
SH-21P	1998/12/31	SH without atypia	2002/12/28	endometrioid	NA	1	QPCR
SH-22P	2002/11/9	SH/CH without atypia	2008/6/19	endometrioid	1A	2	QPCR
SH-27P	2010/2/10	SH without atypia	2015/1/22	endometrioid	IA	1	QPCR
SH-29P	1995/9/16	SH without atypia	2015/7/18	endometrioid	IA	1	QPCR
SH-30P	2007/5/11	SH without atypia	2012/12/5	endometrioid	IIIA	2	QPCR
SH-32P	1993/2/6	SH/CH without atypia	2004/11/29	endometrioid	IIIA	2	QPCR
SH-35P	2002/10/4	SH without atypia	2019/4/25	endometrioid	IA	1	QPCR
SH-36P	2002/11/9	SH without atypia	2020/6/23	endometrioid	IA	1	QPCR
SH-37P	2005/6/17	SH/CH without atypia	2021/1/22	endometrioid	1A	1	QPCR
SH-38P	2017/3/27	SH without atypia	2020/6/23	endometrioid	IA	1	QPCR
SH-39P	2012/7/4	SH without atypia	2021/1/22	endometrioid	IB	1	QPCR

Em. ca.: endometrial cancer; Dx: diagnosis; SH: simple hyperplasia; CH: complex hyperplasia; NA: not available.

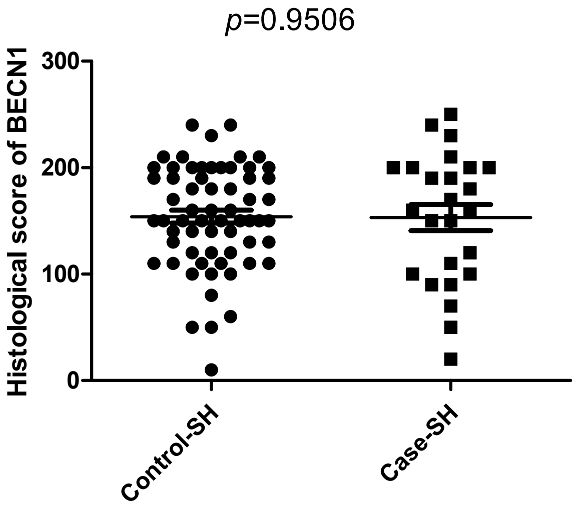

Figure S1. Histoscores of BECN1 in patients with control EH and case EH tissues. The protein levels of BECN1 were analyzed through IHC in the tissue samples from SH/CH-nonA progressed to EC (Case-SH) and those whose did not (Control-SH).

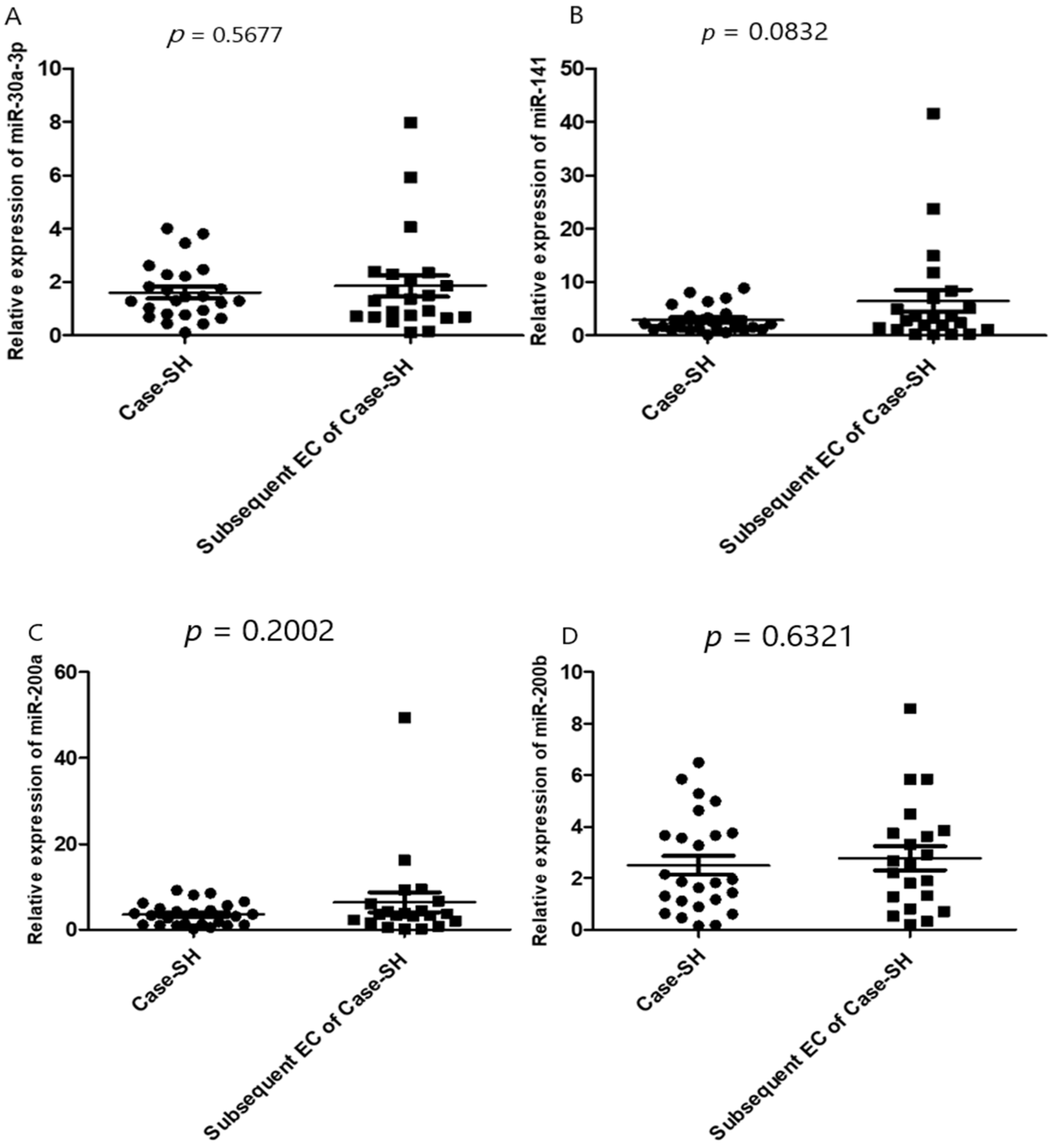

Figure S2. Differential expression of miRNAs in EC tissue and EH tissue in case with SH/CH-nonA. **(A–D)** The expression levels of miR-30a-3p, miR-141, miR-200a and miR-200b were not significantly different between the EH tissue in case with SH/CH-nonA (Case-SH) and EC tissue (Subsequent EC of Case-SH) by using RT-qPCR. The expression of miRNAs was normalized to endogenous control miR-16.

(A)

GO.term.accession	GO.term.name	GO.domain	num_genes	num_intersection	p.value	adj.p.value
GO:0019835	cytolysis	biological_process	20	3	0.000176467485933474	0.365385791674339
GO:0019369	arachidonic acid metabolic process	biological_process	23	3	0.000270846354012866	0.365385791674339
GO:0000096	sulfur amino acid metabolic process	biological_process	5	2	0.000301388571020689	0.365385791674339
GO:0015793	glycerol transport	biological_process	6	2	0.00045044478118883	0.365385791674339
GO:0042426	choline catabolic process	biological_process	6	2	0.00045044478118883	0.365385791674339

(B)

GO.term.accession	GO.term.name	GO.domain	num_genes	num_intersection	p.value	adj.p.value
GO:0070125	mitochondrial translational elongation	biological_process	88	9	0.0000547996588868838	0.168899279325252
GO:0019369	arachidonic acid metabolic process	biological_process	23	5	0.0000694059089070276	0.168899279325252
GO:0009642	response to light intensity	biological_process	7	3	0.000243444898412104	0.394948773523903
GO:0070126	mitochondrial translational termination	biological_process	89	8	0.000348203049183704	0.423676060094272
GO:0032835	glomerulus development	biological_process	9	3	0.00056749367031661	0.552398338686188

Figure S3. A prediction of biological processes targeted by (A) 20 miRNAs or (B) 4 miRNAs by using the data from a website (https://fadeel.shinyapps.io/miRNA-GO-analysis/)
